# SPR965, a Dual PI3K/mTOR Inhibitor, as a Targeted Therapy in Ovarian Cancer

**DOI:** 10.3389/fonc.2020.624498

**Published:** 2021-02-15

**Authors:** Arthur-Quan Tran, Stephanie A. Sullivan, Leo Li-Ying Chan, Yajie Yin, Wenchuan Sun, Ziwei Fang, Sundeep Dugar, Chunxiao Zhou, Victoria Bae-Jump

**Affiliations:** ^1^ Division of Gynecologic Oncology, University of North Carolina at Chapel Hill, Chapel Hill, NC, United States; ^2^ Department of Advanced Technology R&D, Nexcelom Bioscience LLC, Lawrence, MA, United States; ^3^ Department of Gynecologic Oncology, Beijing Obstetrics and Gynecology Hospital, Capital Medical University, Beijing, China; ^4^ Sphaera Pharma Singapore Pte Ltd., Singapore, Singapore; ^5^ Lineberger Comprehensive Cancer Center, University of North Carolina at Chapel Hill, Chapel Hill, NC, United States

**Keywords:** SPR965, cell proliferation, invasion, cell stress, ovarian cancer

## Abstract

SPR965 is an inhibitor of PI3K and mTOR C1/C2 and has demonstrated anti-tumorigenic activity in a variety of solid tumors. We sought to determine the effects of SPR965 on cell proliferation and tumor growth in human serous ovarian cancer cell lines and a transgenic mouse model of high grade serous ovarian cancer (KpB model) and identify the underlying mechanisms by which SPR965 inhibits cell and tumor growth. SPR965 showed marked anti-proliferative activity by causing cell cycle arrest and inducing cellular stress in ovarian cancer cells. Treatment with SPR965 significantly inhibited tumor growth in KpB mice, accompanied by downregulation of Ki67 and VEGF and upregulation of Bip expression in ovarian tumors. SPR965 also inhibited adhesion and invasion through induction of the epithelial–mesenchymal transition process. As expected, downregulation of phosphorylation of AKT and S6 was observed in SPR965-treated ovarian cancer cells and tumors. Our results suggest that SPR965 has significant anti-tumorigenic effects in serous ovarian cancer *in vitro and in vivo*. Thus, SPR965 should be evaluated as a promising targeted agent in future clinical trials of ovarian cancer.

## Introduction

Ovarian cancer represents the second most common gynecologic malignancy and is the fourth most common cause of death for women within the United States (1). Most women who are diagnosed with ovarian cancer are diagnosed with advanced stage disease. Standard treatment for ovarian cancer involves a combination of surgical resection and platinum-based combination chemotherapy. Although this combination of treatment appears to be effective, most women will inevitably develop recurrent disease (2). Therefore, studies have shifted toward targeting specific pathways and cellular targets (3–5), with some success such as anti-angiogenic agents and poly (ADP-ribose) polymerase (PARP) inhibitors.

The phosphatidylinositol-3-kinase (PI3K)/Akt/mammalian target of rapamycin (mTOR) pathway has been implicated in the development of multiple types of malignancies (6–8). This pathway is responsible for the regulation of cellular growth, metabolism, and cell cycle progression (8, 9). PI3KCA, specifically, have been shown to be amplified and overexpressed in cases of high grade serous ovarian cancer (10, 11), while AKT and mTOR activation have been observed frequently in clear cell ovarian carcinoma (12). Moreover, high expression and mutation of the PI3K/AKT/mTOR pathways have shown to be a poor prognostic factor for patients with ovarian cancer (11, 13).

There are multiple trials investigating AKT and mTOR inhibitors in patients with ovarian cancer. Several AKT inhibitors in phase I or II clinical trials were tolerable and in combination with chemotherapeutic agents showed evidence of clinical activity in ovarian cancer (14–17). However, phase II trials of temsirolimus, a single agent mTOR inhibitor, have shown little activity in patients with persistent or recurrent ovarian cancer (18, 19). Although results from phase II trials of single agent inhibitors are discouraging, inhibition of a single pathway within the complex PI3K/AKT/mTOR network may not be effective. Currently, pre-clinical data shows that the dual inhibitors, CMG002 and BEZ235, may be more effective in inhibiting ovarian cancer cell proliferation and tumor growth (20, 21).

SPR965 is an orally available, novel small molecule that is an inhibitor of PI3K and mTOR C1/C2 kinases with favorable linear pharmacokinetics profiles and microsomal stability (22, 23). The oral bioavailability of SPR965 in Sprague Dawley rats and in nude mice was ∼100% and 75%, respectively. SPR965 exhibits anti-proliferative activity in multiple cell lines and in several xenograft models including multiple myeloma, prostate, and colon cancer (22, 23). Therefore, we sought to investigate the effect of SPR965 on cell proliferation and tumor growth in human serous ovarian cancer cells and a transgenic mouse model of high grade serous ovarian cancer.

## Materials and Methods

### Cell Culture and Reagents

Four ovarian cancer cell lines, SKOV3, Hey, OVCAR433, and OVCAR5, were used. The SKOV3, OVCAR5 and OVCAR433 cells were grown in DMEM/F12 medium supplemented with 10% bovine serum. The Hey cell line was maintained in RPMI 1640 medium supplemented with 5% bovine serum, 100 units/ml penicillin, and 100 microgram/ml streptomycin under 5% CO_2_.

SPR965 was obtained from Sphaera Pharma (Singapore). The Annexin V FITC kit was purchased from BioVision (Mountain View, CA). All antibodies were purchased from Cell Signaling (Beverly, MA). Enhanced chemiluminescence western blotting detection reagents were purchased from Thermo (Rockford, IL). All other chemicals were purchased from Sigma (St. Louis, MO).

### MTT Assay

The SKOV3, Hey, OVCAR433, and OVCAR5 cells were plated and grown in 96-well plates at a concentration of 4,000 cells/well for 24 h. The cells were subsequently treated with varying doses of SPR965 for 72 h. MTT (5 mg/ml) was added to the 96-well plates at 5 μl/well, followed by an additional hour of incubation. The MTT reaction was terminated through the addition of 100 μl of 100% DMSO. The results were read by measuring absorption at 575 nm with a microplate reader (Tecan, Morrisville, NC). The effect of SPR965 was calculated as a percentage of control cell growth obtained from DMSO treated cells grown in the same 96-well plates. Each experiment was performed in triplicate to assess for consistency of results.

### Cell Proliferation Assay Using Image Cytometry

The cell proliferation assay was performed using the Celigo Image Cytometer. First, the SKOV3 and HEY target cells were seeded in a 96-well plate (Greiner 655090) at 5,000 cells/well. After overnight incubation, the cells were treated with the SPR965 compound at 0.1, 1, 10, 100, 500, 1,000, 5,000, and 10,000 nM, including a media control (n = 4 for each condition). The plate was imaged in bright field and analyzed using the image cytometer at t = 0, 6, 24, 48, and 72 h post-treatment. The confluence percentages of the target cells at each time point were measured from the bright field images to generate the time-course data. In addition, the end point confluence percentages at 72 h were collected to generate dose response curves for both cell types. All experiments were performed at least twice to assess for consistency of results.

### Cell Cycle Assay Using Image Cytometry

The cell cycle assay was performed using the Celigo Image Cytometer. First, the SKOV3 and HEY cells were seeded into two 96-well plate (Greiner 655090) at 10,000 cells/well for 24 and 36 h of subsequent drug treatment. After overnight incubation, the cells were treated with the SPR965 compound at 1, 100, 500, and 1,000 nM, including a media control (n = 4 for each condition). After 24 and 36 h of treatment with SPR965, the 96-well plates were stained using the ViaStain™ PI Cell Cycle Kit for Celigo (CS1-V0004-1, Nexcelom Bioscience, Lawrence, MA), following the manufacturer’s protocol. The plates were then imaged in both bright field and red fluorescence channels to measure the propidium iodide (PI) fluorescent signals. The fluorescent intensity data were then exported into FCS Express (De Novo Software, Pasadena, CA) to generate cell cycle plots for automated determination of G0/G1, S, and G2/M cell population percentages. All experiments were performed at least twice to assess for consistency of results.

### Apoptosis Assay Using Image Cytometry

The apoptosis assay using caspase 3/7 fluorescence labeling was performed using the Celigo Image Cytometer. First, the SKOV3 and HEY target cells were seeded into 96-well plates (Greiner 655090) at 10,000 cells/well for 6 and 12 h of drug treatment. After a 2-day incubation, the cells were treated with the SPR965 compound at 1, 100, 500, and 1,000 nM, including a media control (n = 4 for each condition). After 6 and 12 h of drug treatment, the 96-well plates were fixed and stained using the ViaStain™ Live Caspase 3/7 Detection for 2D/3D Culture with Hoechst (CSK-V0003-1, Nexcelom Bioscience), following the manufacturer’s protocol. The plates were then imaged in bright field, blue, and green fluorescence channels to identify the target cells with Hoechst fluorescence and measure the Caspase 3/7 green fluorescent signals. The fluorescent intensity data was directly plotted in the Celigo software to generate quadrant plots to determine the caspase 3/7 positive cell populations. All experiments were performed at least twice to assess for consistency of results.

### Reactive Oxygen Species (ROS) Assay

The alteration of total production of reactive oxygen species caused by SPR965 was measured using a DCFH-DA fluorescent dye. The SKOV3 and Hey cells (5000 cells/well) were seeded in black 96-well plates. After 24 h, the cells were treated with SPR965 for 8 h to induce ROS generation. After the cells were incubated with DCFH-DA (20 μM) for 30 min, the fluorescence was monitored at an excitation wavelength of 485 nm and an emission wavelength of 530 nm using a Tecan plate reader. All experiments were performed at least twice to assess for consistency of results.

### Western Immunoblotting

The SKOV3 and Hey cells were plated at 2.5 × 10^5^ cells/well in 6-well plates in their corresponding media and then treated with SPR965 overnight. Cell lysates were prepared in RIPA buffer. Equal amounts of protein were separated by gel electrophoresis and transferred onto a PVDF membrane. The membrane was blocked with 5% nonfat dry milk and then incubated with a 1:1,500 dilution of primary antibody overnight at 4°C. The membrane was then washed and incubated with a secondary peroxidase-conjugated antibody for 1 h after washing. Antibody binding was detected using an enhanced chemiluminescence detection system on the Alpha Innotech Imaging System (Protein Simple, Santa Clara, CA). After developing, the membrane was stripped and re-probed using antibodies against α-Tubulin to confirm equal loading. Intensity for each band was measured and normalized to α-Tubulin as an internal control. Each experiment was repeated two times to assess for consistency of results.

### Genetically Engineered Mouse Model of Ovarian Cancer

The ${\text{K}18-\text{gT}}_{121}^{+/-}$;p53^fl/fl^;Brca1^fl/fl^ (KpB) mouse model of ovarian cancer were used (24, 25). All experimental mice were maintained in accordance with the Institutional Animal Care and Use Committee (IACUC) and the NIH guide for the Care and Use of Laboratory Animals. Recombinant adenovirus Ad5-CMV-Cre (AdCre) was purchased from the University of Iowa Transfer Vector Core at a titer of 10^11^–10^12^ infectious particles/ml. The AdCre was then injected *via* a needle introduced into the oviduct near the infundibulum and into the ovarian bursa to induce ovarian cancer at 6–8 week of age. The KpB mice were monitored weekly by palpation for tumor growth. SPR965 and placebo treatment was initiated after palpation of a 0.1–0.2 cm tumor in mice. The mice were randomly divided into two groups (15 mice/per group) including control and SPR965 treatment groups. The mice were treated with SPR965 (3 mg/kg, oral gavage) every 5 days for 4 weeks, All mice were euthanized after 4 weeks of SPR965 treatment. Ovarian tumor tissues were collected for immunohistochemical staining.

### Immunohistochemical Staining (IHC)

Ovarian tumor tissues from mice were formalin-fixed and paraffin-embedded. After rehydration and antigen retrieval, the slides (thickness = 4 μm) were incubated with primary antibodies: anti-Ki-67, anti-Bip, anti-phos-S6, anti-phos-AKT and anti-VEGF. The staining was visualized using DAB (Invitrogen, CA). The slides were scanned by Motic (Warrendale, PA). The expression analysis of Ki67, Bip, phos-S6, phos-AKT and VEGF were extracted using the following Image-Pro software (Rockville, MD). Images of the areas of expression were selected for clarity from at least five fields for each IHC specimen. The positive cells were detected, quantified and averaged automatically in selected areas. Each group was measured in seven IHC slides.

### Statistical Analysis

Data are given as the mean ± SD. Statistical significance was analyzed by the two-sided unpaired Student’s t-test from at least three replicates. Tumor growth in different treatment arms was analyzed by One-way & Two-way ANOVA test. GraphPad Prism 6 (La Jolla, CA USA) was used for all graphs and significance tests. P-values of < 0.05 were considered to have significant group differences.

## Results

### SPR 965 Inhibits Cell Proliferation in Ovarian Cancer Cell Lines

**Figure 1:**
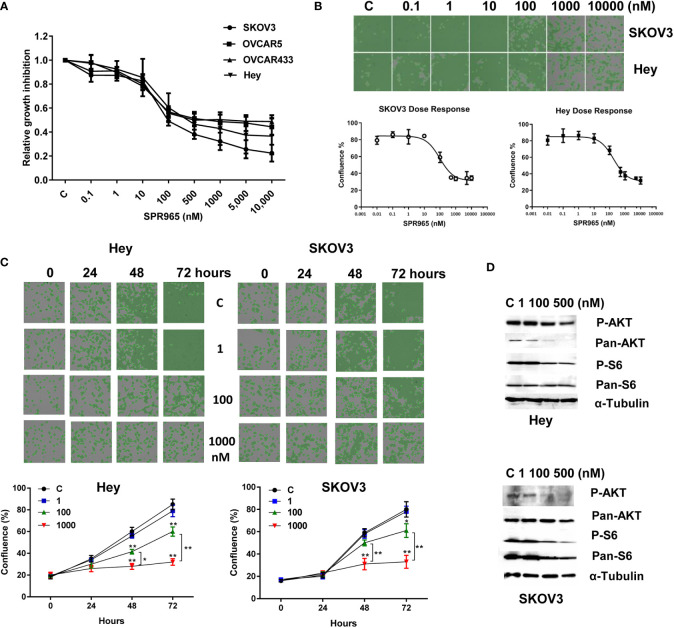
SPR965 induced dose-dependent growth inhibition in ovarian cancer cells. The OVCAR5, OVCAR433, Hey, and SKOV3 cell were treated with various doses of SPR965 for 72 h. Cell proliferation was determined by MTT assay **(A)** Celigo Image Cytometer was used to determine the effect of SPR965 on cell proliferation in SKOV3 and Hey cells **(B)**. Relative survival was determined by dividing the number of remaining DHA treated cells by the number of remaining viable DMSO. Treatment of the cells with SPR965 at 1, 100, and 1,000 nM for 24, 48, and 72 h showed that SPR965 induced inhibition of cell proliferation in time dependent manner in both cell lines **(C)**. The images are one representative result of three independent experiments. Western blotting results showed that SPR965 reduced the expression of phosphorylated-AKT and phosphorylated-S6 in the Hey and SKOV3 cells **(D)**. *p < 0.05; **p < 0.01.

The effects of SPR965 on ovarian cancer cell proliferation were investigated using the MTT assay and the image cytometry-based cell counting assay. The SKOV3, Hey, OVCAR433 and OVCAR5 cells were treated with SPR965 at varying doses for 72 h. MTT assay showed that SPR965 significantly displayed cytotoxicity against four ovarian cancer cell lines in a dose dependent manner with IC_50_ values in the ranges of 110 nM in SKOV3, 390 nM in Hey, 490 nM in OVCAR433 and 750 nM in OVCAR5, respectively (**Figure 1A**). Similarly, image cytometry-based cell counting assay also showed that SPR965 reduced cell counts in a dose-dependent manner after treatment of 72 h in Hey and SKOV3 cells and both cell lines exhibited similar drug responses to SPR965 (**Figure 1B**). Additionally, we performed a time-course experiment to determine the inhibitory effect of SPR965 in the Hey and SKOV3 cells treated with doses of 1, 100, and 1,000 nM. The results showed that the inhibitory effects of SPR965 were significant at 12 h of exposure in the SKOV3 cells and at 24 h in the Hey cells. As treatment doses increased, cell counts decreased over time compared to controls (**Figure 1C**).

Given that SPR965 is a dual inhibitor of PI3K and mTOR C1/C2 kinases, we investigated the effect of SPR965 on the expression of phosphorylated AKT and S6 in the SKOV3 and Hey cells. Both cell lines were treated with SPR965 at doses of 1, 100, and 500 nM for 24 h. As expected, western blotting results confirmed that the level of phosphorylated AKT and S6 was markedly inhibited by SPR965 in both cells (**Figure 1D**). These results suggest that SPR965 can effectively inhibit cell growth *via* inhibition of AKT/mTOR pathways in ovarian cancer cells.

### SPR 965 Induces G1 Phase Cell Cycle Arrest But Not Apoptosis

**Figure 2:**
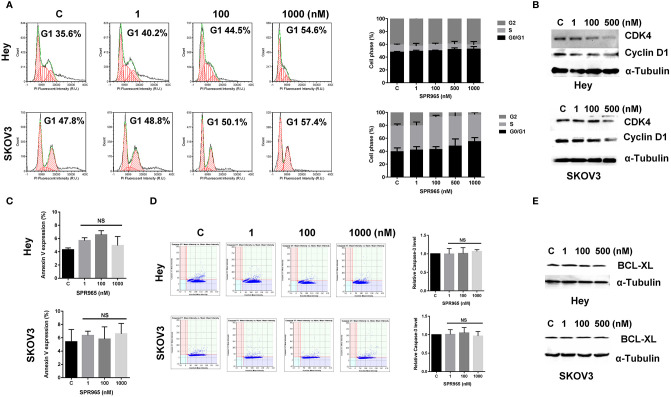
Effect of SPR965 on cell cycle and apoptosis in ovarian cancer cells. The Hey and SKOV3 cells were treated with the indicated doses of SPR965 for 24 h. Cell cycle progression was assessed by Celigo Image Cytometer. SPR965 induced cell cycle arrest in G1 phase in the Hey and SKOV3 cells **(A)**. Cell lysates from the Hey and SKOV3 cells were subjected to Western blotting analysis for the expression of CDK4 and Cyclin D1 **(B)**. The results from Celigo Image Cytometer showed that SPR965 did not increase the expression of Annexin V or the activity of cleaved caspase 3/7 in either cell line **(C, D)**. The figures represented three independent experiments. Western blotting analysis demonstrated that SPR965 did not affect the expression of BCL-XL in the Hey and SKOV3 cells after 24 h of treatment **(E)**. Data are representative of one of three repeats *p < 0.05 and **p < 0.01.

To evaluate the underlying mechanism of cell proliferation inhibition by SPR965, the cell cycle profile was analyzed by the image cytometer after treating the Hey and SKOV3 cell lines with varying doses of SPR965 for 24 h. As illustrated in **Figure 2A**, SPR965 induced G1 phase cell cycle arrest and reduced S phase in a dose-dependent manner. Treatment of the cells with SPR965 at 1 μM increased cells in G0/G1 phase from 35.6% to 54.6% in Hey cells and 47.8% to 57.4% in SKOV3 cells compared to controls (p < 0.05). Western blotting results showed that SPR965 reduced the expression of CDK4 and cyclin D1 in a dose-dependent manner after 24 h of treatment in both cell lines (**Figure 2B**).

We next investigated the effect of SPR965 on apoptosis using the image cytometer in the SKOV3 and Hey cells. Both cell lines were treated with SPR965 at varying concentrations for 12, 24, and 48 h. The results showed that SPR965 had no effect on Annexin V expression in both cell lines (**Figure 2C**). Treatment of both cell lines with SPR965 for 6 and 12 h did not increase the expression of cleaved caspase 3/7 (**Figure 2D**). Furthermore, SPR965 did not alter BCL-XL expression in the Hey and SKOV3 cells after treatment for 24 h (**Figure 2E**). These results suggest that SPR965 inhibits cell growth through cell cycle G1 phase arrest, but does not impact apoptosis.

### SPR 965 Inhibits Tumor Growth in KpB Mouse Model

**Figure 3:**
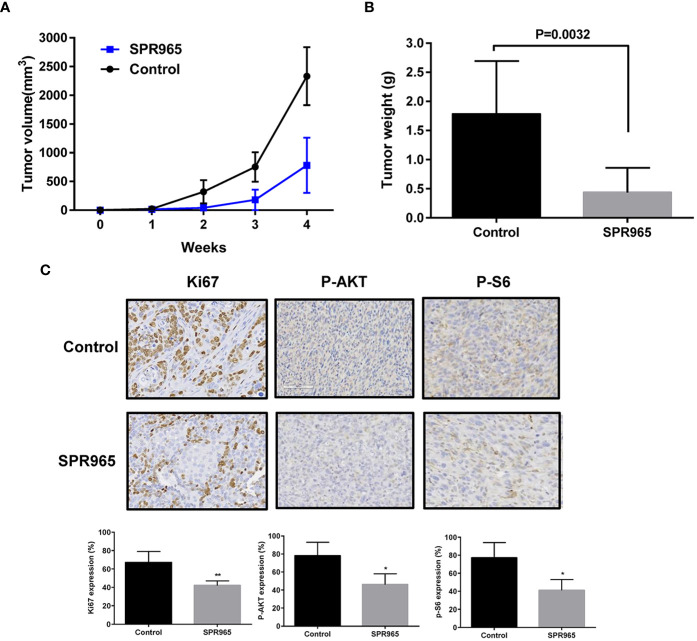
SPR965 inhibited tumor growth in KpB mice. KpB mice were treated with vehicle (control) or SPR965 (3 mg/kg, oral gavage) for 4 weeks when the tumors reached 0.1–0.2 cm in size. SPR965 significantly inhibited tumor volume and tumor weight (N=15 animals per group) as compared to the vehicle treated controls **(A, B)**. Immunohistochemistry results showed that SPR965 decreased the expression of Ki-67, phos-S6, and phos-AKT in the ovarian tumor tissues **(C)**. *p < 0.05; **p < 0.01.

To validate the anti-tumorigenic potential of SPR965 *in vivo*, we utilized a transgenic model for high grade serous ovarian cancer (KpB mice). When tumors reached a size of 0.1–0.2 cm in diameter, mice were treated with SPR965 (3 mg/kg/day, oral gavage) or vehicle (saline) for 4 weeks. Tumor growth during treatment was monitored by palpation and calipers twice a week. At the end of treatment, the mice were euthanized, and the ovarian tumors were removed, photographed, and weighed. During the treatment, SPR965-treated mice tolerated the treatment well; mice showed no changes in body weight and were able to maintain their normal activity. SPR965 significantly reduced tumor volume and inhibited tumor weight compared to the control group (**Figures 3A, B** **)**. Ovarian tumor weight decreased by 75.26% with SPR965 treatment when compared with control mice (p < 0.01).

To further confirm the anti-tumorigenic activity and mechanism of action of SPR965 in KpB mice, the expression of Ki-67, phosphorylated-AKT and phosphorylated–S6 was evaluated by immunohistochemistry (IHC). Consistent with our results *in vitro*, SPR965 significantly inhibited Ki-67 expression in the SPR965-treated mice by 24.6% (p < 0.01). As expected, we also found that the expression of phosphorylated-AKT and phosphorylated-S6 was decreased in the KpB mice treated with SPR965 (**Figure 3C**). These results suggest that SPR965 inhibits ovarian tumor growth through inhibition of AKT/mTOR pathway *in vivo*.

### SPR 965 Induces Cellular Stress

**Figure 4:**
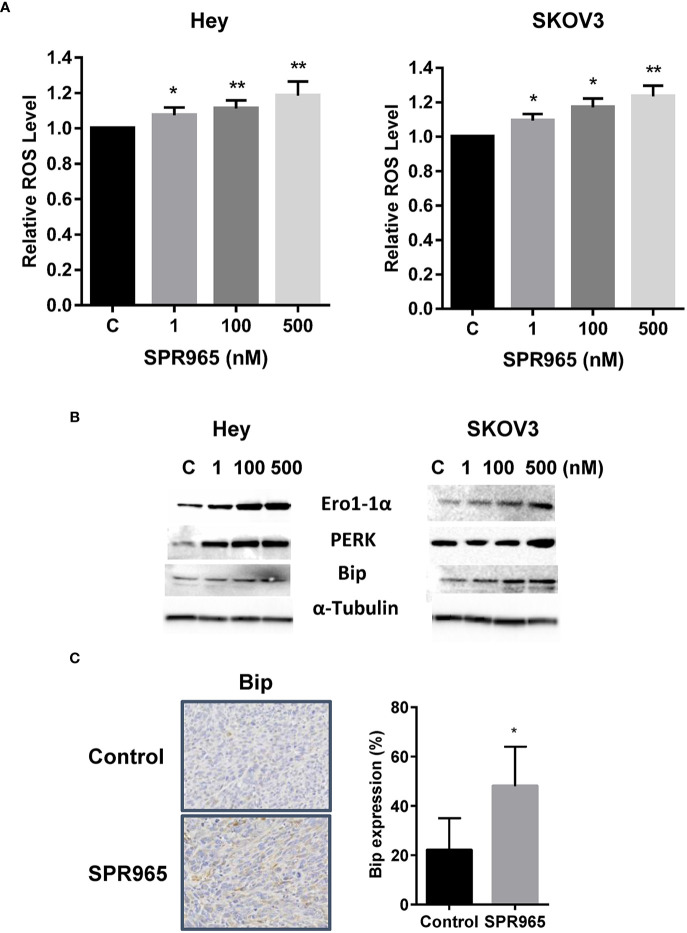
SPR965 induced endoplasmic reticulum (ER) stress in ovarian cancer cells. The Hey and SKOV3 cells were treated with SPR965 at different concentrations for 8 h. Reactive oxygen species (ROS) products were measured by DCFH-DA assay. SPR965 significantly increased the levels of ROS in both cell lines compared to the control cells **(A)**. Western blotting results showed that SPR965 increased the expression of cellular stress-related proteins including PERK, Ero1, and Bip in both cell lines after treatment for 24 h **(B)**. Immunohistochemistry results showed that SPR965 increased the expression of Bip in the ovarian tumors of KpB mice **(C)**. The results are shown as the mean ± SD and are representative of three independent experiments. *p<0.05, **p<0.01.

To investigate the involvement of oxidative stress on the anti-proliferative effects of SPR965, intracellular ROS levels were examined using ROS fluorescence indicator DCF-DA. The SKOV3 and Hey cells were treated with SPR96 at different doses for 8 h. SPR965 significantly increased ROS production in a dose-dependent manner in both cell lines. At a concentration of 500 nM, SPR965 significantly increased the DCFH-DA fluorescence by 18.5% and 23.5% in the Hey and SKOV3 cells, respectively (**Figure 4A**, p < 0.01). Since Bip is a master endoplasmic reticulum (ER) regulator which activates PERK, leading to increased ROS production and ERO1 activity under abnormal conditions (26), we next analyzed the response of ER stress-related markers after treatment with SPR965 for 24 h in the Hey and SKOV3 cells. Western blot results revealed that SPR965 significantly induced the protein expression of PERK, Bip, and Ero1 in a dose-dependent manner in both cell lines. Furthermore, our immunohistochemistry results showed that Bip expression was increased by 26.1% in SPR965 treated ovarian tumors compared to control mice (**Figure 4C**, p < 0.05). These results indicate that an increase in ROS production and ER stress might also be involved in the anti-proliferative and anti-tumorigenic effects of SPR965 on ovarian cancer.

### SPR 965 Reduces Cellular Invasion

**Figure 5:**
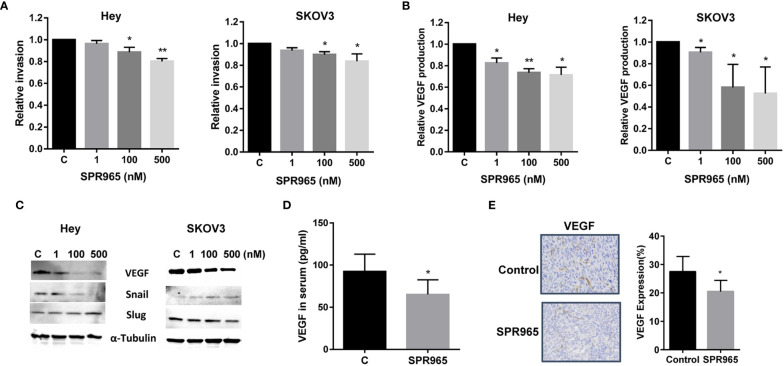
SPR965 inhibited invasion in ovarian cancer cells. SPR965 inhibited cell invasion in the Hey and SKOV3 cells, assessed by transwell assays **(A)**. The Hey and SKOV3 cells were treated with SPR965 for 24 h. VEGF products in the media were assessed by VEGF ELISA assay **(B)**. Western blotting results indicated that SPR965 affected the expression of epithelial-mesenchymal transition (EMT) markers including an increase in Slug in Hey cells and a decrease in Snail in both cell lines **(C)**. SPR965 reduced serum VEGF levels in SPR965-treated KpB mice at the end of treatment **(D)**. Immunohistochemistry results confirmed that SPR965 significantly reduced the expression of VEGF in ovarian tumors of KpB mice **(E)**. *P < 0.05, **P<0.01.

In order to determine the effect of SPR965 on invasion of ovarian cancer cells, an *in vitro* transwell invasion system was employed. The Hey and SKOV3 cells were seeded into the upper chambers of the transwell and treated with SPR965 (1–500 nM) for 3 h. The invasive capacity of the Hey and SKOV3 cell lines was reduced by SPR965 treatment in a dose-dependent manner. SPR965 (500 nM) significantly reduced the invasive ability of the Hey and SKOV3 cell lines by 17.8% and 16.4% (**Figure 5A**, p < 0.01), respectively. We next measured VEGF productions in cell culture media of Hey and SKOV3 after treatment with SPR965 for 24 h. VEGF ELISA assay showed that SPR965 greatly reduced media VEGF levels in both cell lines. Treatment of cells with 100 nM SPR965 decreased VEGF production by 27.6% in the Hey and 46.2% in the SKOV3 cells (**Figure 5B**).

To further analyze the effect of SPR965 on epithelial-mesenchymal transition (EMT) in ovarian cancer cell lines, the expression of VEGF, Snail, and Slug were analyzed by Western immunoblotting. As expected, SPR965 decreased the expression of VEGF and Snail in both cell lines. However, SPR965 increased the expression of Slug in Hey cells and reduced Slug expression in SKOV3 cells, suggesting that the role of SPR965 in inhibition of cell invasion may be involved in different steps on EMT depending on the cell line (**Figure 5C**). Furthermore, serum levels of VEGF in KpB mice were found to be significantly decreased by 29.5% in SPR965-treated mice compared to control group (**Figure 5D**, p < 0.05). We also found that the expression of VEGF protein was decreased by 17.4% in SPR965-treated ovarian tumors in comparison to placebo-treated mice, as detected by immunohistochemistry (**Figure 5E**), p < 0.05). These results indicate that the inhibition of cell invasion by SPR965 may involve both EMT processes and angiogenesis in ovarian cancer.

## Discussion

Activation of the PI3K/Akt/mTOR pathway in ovarian cancer has been associated with carcinogenesis and progression (27, 28). Activation of the PI3K/Akt signal pathway is also well known to cause drug resistance to chemotherapy (10, 12, 13). This pathway is complex with multiple feedback loops which has made targeting this pathway challenging. For instance, downregulation of mTOR has been associated with an increase in AKT phosphorylation (29). Small molecule inhibitors have been investigated in ovarian cancer but have demonstrated limited clinical efficacy. The small molecule inhibitors, ARQ 092 and ARQ 087, through AKT inhibition cause G1 cell cycle arrest without apoptotic effects; however, clinical significance was not noted in patients with ovarian cancer (30). Additionally, the rapamycin analogs, temsirolimus and everolimus, downregulate the mTOR pathway; however, clinical responses have been modest (18, 19, 31). The lack of significant clinical response can likely be attributed to the complex nature of the PI3K/AKT/mTOR pathway and multiple feedback loops whereas inhibition at one site may upregulate other regulatory proteins (32, 33). Thus, dual inhibition of PI3K and mTOR has the advantage to overcome the upregulation of PI3K due to the feedback loop caused by mTOR inhibition, which may improve treatment response for ovarian cancer (33, 34).

It has been shown that the use of PI3K and mTOR inhibitors in combination leads to increased inhibition of cancer cell proliferation and tumor growth. Data from two other small molecule dual inhibitors show that dual inhibition of the PI3K/AKT/mTOR pathway suppresses cell proliferation, induces G1 cell cycle arrest, and induces apoptosis in ovarian cancer cells and mouse models (21, 35). Our study is the first to investigate the novel AKT/mTOR dual inhibitor, SPR965, in the setting of serous ovarian cancer. Our results mirror that of other dual inhibitors by decreasing cell proliferation, causing G1 cell cycle arrest and inducing cellular stress. SPR965 specially reduced the expression of phosphorylation of AKT and S6 and significantly inhibited tumor growth in our KpB mice accompanied by decreased levels of Ki-67, VEGF, phosphylated-AKT and phosphylated-S6 protein expression, as well as increased the expression of Bip in tumor tissues. Furthermore, we have confirmed that SPR965 was able to reduce the ability of adhesion and invasion in ovarian cancer cells and ovarian tumors of KpB mice.

Cell cycle arrest and apoptosis are common mechanisms responsible for the inhibition of cancer cell proliferation and tumor growth (36, 37). Many studies have identified that the PI3K/AKT/mTOR pathway is critical in control cell cycle in cancer cells. Most of the dual AKT/mTOR inhibitors significantly led to cell cycle G1 arrest and apoptosis (38, 39). However, several reports suggest that apoptosis has a controversial role in the anti-cancer effect of dual AKT/mTOR inhibitors (40). Ant-proliferative activity of the dual AKT/mTOR inhibitor, BEZ235, correlated with a dose-dependent increase of gastric cancer cells in the G1 phase of the cell cycle and cyclin D1 downregulation (41). Dual inhibition of the PI3K/AKT/mTOR pathway with MK2206 and BEZ235 caused cell cycle arrest and enhanced apoptosis through the downstream effectors SKP2, MCL-1, and cyclin D1 in esophageal squamous cell carcinoma cells. MK2206/BEZ235 in combination showed greater anti-tumorigenic effects than MK2206 or BEZ235 alone in xenograft mice (42). The PI3K/mTOR dual inhibitor GSK458 showed inhibitory efficacy against ovarian cancer cell proliferation and tumor growth through induction of cell cycle G1 arrest in nude mice and a PDX model (43). In our present study, SPR965 treatment caused significant cell cycle G1 phase arrest but failed to induce apoptosis in ovarian cancer cells. Furthermore, we found no difference in expression of cleaved caspase 3 in SPR965 treated mice compared to control mice (data not shown). Thus, we conclude that SPR965 inhibited cell proliferation predominantly through induction of cell cycle arrest in ovarian cancer cells as opposed to have any effect on apoptosis.

The activation of the PI3K/AKT/mTOR pathway promotes the expression of genes related to cell proliferation, invasion, and migration in ovarian cancer. Inhibition of the AKT/mTOR pathway by the dual inhibitors, GSK458, AZD2014 and BEZ235 reduced cell invasion and migration in thyroid, pancreatic, and ovarian cancer in preclinical models (43–46). A recent study has investigated the effect of AZD2014 and BEZ235 in invasion in comparison with single AKT or mTOR inhibitors in bladder cancer cells. AZD2014 and BEZ235 exerted stronger inhibitory effects on cancer cell invasion and EMT-related signaling pathways and mesenchymal markers compared to the single agents alone (47). In ovarian cancer, BEZ235 significantly prevents hypoxia- and TGF-β1-induced EMT and up-regulates the E-cadherin expression *in vitro* and *in vivo*, suggesting that dual inhibition of AKT and mTOR may have the potential for treatment of cancer metastasis (48). Our results demonstrated that SPR965 inhibited cell invasion through reduction of EMT processes and reduced serum VEGF production and expression of VEGF in ovarian tumors in KpB mice, indicating that SPR965 may be a promising agent in the prevention of ovarian cancer metastasis.

Recently, targeting of AKT/mTOR with dual inhibitors has been described to induce oxidative stress as well as expression of oxidative stress related to proteins. BEZ235 triggered strong ROS production in cholangiocarcinoma and ovarian cancer cells, which is associated with induction of apoptosis (49, 50). In addition to stimulating apoptosis and autophagy, ROS and oxidative stress may play an important role in controlling certain stages of the cell cycle in cancer cells. ROS has been confirmed to initiate checkpoint arrest and induce a G1 checkpoint response (51). We here observed that SPR965 increased ROS production and the expression of PERK, Erol-1 and Bip in the Hey and SKOV3 cells. Immunochesmistry showed that SPR965 increased Bip expression in ovarian tumors in KpB model. Therefore, cell cycle G1 arrest and oxidative stress as opposed to inducing apoptosis may be the most critical mechanisms for the anti-tumorigenic effect of SPR965 in ovarian cancer. In addition, autophagy induced by SPR965 is currently being investigated in our lab.

## Conclusions

Our study found that SPR965 inhibited cell proliferation and tumor growth by inducing cell cycle G1 arrest and oxidative stress *via* blocking the PI3K/Akt/mTOR pathway in ovarian cancer. Moreover, SPR965 decreased cell invasion through EMT processes in ovarian cancer cells, and reduced VEGF levels in serum and ovarian tumors in a transgenic mouse model of ovarian cancer. Taken together, our findings provide valuable insight into the underlying biology behind the actions of SPR965 as a dual inhibitor of the AKT/mTOR pathway, and highlight the potential promise of this agent in the treatment of highly lethal ovarian cancer.

## Data Availability Statement

The original contributions presented in the study are included in the article/supplementary materials. Further inquiries can be directed to the corresponding authors.

## Ethics Statement

The animal study was reviewed and approved by Institutional Animal Care and Use Committee, UNC. Written informed consent was obtained from the owners for the participation of their animals in this study.

## Author Contributions

A-QT, SS, YY, WS, and ZY performed the experiments *in vitro*. ZY and WS treated the mice with SPR965. LC performed and analyzed the experiments of image cytometry, performed the image cytometric assay and analysis for the cell proliferation, cell cycle, and apoptosis assays. A-QT, SS, CZ, and VB-J participated in analyzing and interpreting the data. A-QT, CZ, and VB-J wrote the manuscript. SD provided SPR965. CZ and VB-J designed experiments, revised the manuscript, and provided financial support. All authors contributed to the article and approved the submitted version.

## Funding

This work is supported by (1) VLB: American Cancer Society (ACS) Research Scholar Grant RSG CCE 128826. (2) VLB: NIH/NCI R37CA226969.

## Conflict of Interest

SD is a founder of Sphaera Pharma Singapore Pte Ltd. Sphaera supplied drug for this study. L-YC is an employee of Nexcelom Bioscience LLC.

The remaining authors declare that the research was conducted in the absence of any commercial or financial relationships that could be construed as a potential conflict of interest.
